# Natural Compounds: A Hopeful Promise as an Antibiofilm Agent Against *Candida* Species

**DOI:** 10.3389/fphar.2022.917787

**Published:** 2022-07-11

**Authors:** Aref Shariati, Mojtaba Didehdar, Shabnam Razavi, Mohsen Heidary, Fatemeh Soroush, Zahra Chegini

**Affiliations:** ^1^ Molecular and Medicine Research Center, Khomein University of Medical Sciences, Khomein, Iran; ^2^ Department of Medical Parasitology and Mycology, Arak University of Medical Sciences, Arak, Iran; ^3^ Microbial Biotechnology Research Center, Iran University of Medical Sciences, Tehran, Iran; ^4^ Department of Microbiology, School of Medicine, Iran University of Medical Sciences, Tehran, Iran; ^5^ Department of Laboratory Sciences, School of Paramedical Sciences, Sabzevar University of Medical Sciences, Sabzevar, Iran; ^6^ Cellular and Molecular Research Center, Sabzevar University of Medical Sciences, Sabzevar, Iran; ^7^ Student Research Committee, Khomein University of Medical Sciences, Khomein, Iran; ^8^ Department of Microbiology, School of Medicine, Hamadan University of Medical Sciences, Hamadan, Iran

**Keywords:** natural compounds, *Candida* biofilm, new treatment, essential oil, antibiobilm

## Abstract

The biofilm communities of *Candida* are resistant to various antifungal treatments. The ability of *Candida* to form biofilms on abiotic and biotic surfaces is considered one of the most important virulence factors of these fungi. Extracellular DNA and exopolysaccharides can lower the antifungal penetration to the deeper layers of the biofilms, which is a serious concern supported by the emergence of azole-resistant isolates and *Candida* strains with decreased antifungal susceptibility. Since the biofilms’ resistance to common antifungal drugs has become more widespread in recent years, more investigations should be performed to develop novel, inexpensive, non-toxic, and effective treatment approaches for controlling biofilm-associated infections. Scientists have used various natural compounds for inhibiting and degrading *Candida* biofilms. Curcumin, cinnamaldehyde, eugenol, carvacrol, thymol, terpinen-4-ol, linalool, geraniol, cineole, saponin, camphor, borneol, camphene, carnosol, citronellol, coumarin, epigallocatechin gallate, eucalyptol, limonene, menthol, piperine, saponin, α-terpineol, β–pinene, and citral are the major natural compounds that have been used widely for the inhibition and destruction of *Candida* biofilms. These compounds suppress not only fungal adhesion and biofilm formation but also destroy mature biofilm communities of *Candida*. Additionally, these natural compounds interact with various cellular processes of *Candida*, such as ABC-transported mediated drug transport, cell cycle progression, mitochondrial activity, and ergosterol, chitin, and glucan biosynthesis. The use of various drug delivery platforms can enhance the antibiofilm efficacy of natural compounds. Therefore, these drug delivery platforms should be considered as potential candidates for coating catheters and other medical material surfaces. A future goal will be to develop natural compounds as antibiofilm agents that can be used to treat infections by multi-drug-resistant *Candida* biofilms. Since exact interactions of natural compounds and biofilm structures have not been elucidated, further *in vitro* toxicology and animal experiments are required. In this article, we have discussed various aspects of natural compound usage for inhibition and destruction of *Candida* biofilms, along with the methods and procedures that have been used for improving the efficacy of these compounds.

## Introduction

The incidence of fungal infections, and therapeutic resistance of fungi, is increasing worldwide, intensifying the need for new and more effective antifungal strategies (El-Baz et al., 2021). Candidiasis, caused by various *Candida* species, is an important and life-threatening infection through which *Candida* can enter the bloodstream and host organs during invasive infections (Achkar and Fries, 2010). Worldwide, these fungi rank fifth in causing hospital-acquired infections and are responsible for 50% of catheter-related deaths. *Candida albicans* is the most commonly isolated strain from clinical samples (Brown et al., 2012). Other *Candida* species such as *Candida tropicalis*, *Candida parapsilosis*, *Candida glabrata*, and *Candida krusei* can also cause invasive infections with a high mortality rate in humans (Pappas et al., 2018).

The ability of *Candida* cells to form biofilms and produce hydrolytic enzymes, such as phospholipases and hemolysins, plays an important role in the pathogenesis and drug resistance (Mba and Nweze, 2020). Biofilms are easily formed on the objects that enter the body, such as intravascular and urinary catheters, artificial valves, intrauterine devices, contact lenses, and other foreign objects, or on host surfaces. Biofilm formation begins with the attachment of *Candida* to the surface of a medical device or host (Doke et al., 2014). After the attachment, the yeast is transformed from the planktonic to hyphal state, and the biofilm formation and invasion begin (Sudbery et al., 2004; Finkel and Mitchell, 2011). The extracellular matrix of a biofilm consists of exopolymeric materials that have a protective role against various toxic compounds, such as antifungals, and which also protect the fungal cells from the host immune system (Gupta et al., 2018).

Increased use of catheters and medical implant devices along with biofilm formation, in addition to negatively affecting their performance, leads to persistent infections with the emergence of widespread antifungal resistance (Ramage et al., 2006). Furthermore, many antifungal drugs are currently associated with limitations such as increased resistance, narrow antifungal spectrum, toxicity, and high treatment costs for patients (Lone and Ahmad, 2020). Thus, due to the emergence of multidrug-resistant *Candida* and need for treating its resistant forms, such as biofilms, the application of natural products as new and effective therapies has gained a special place in clinical treatments (Ahmad et al., 2015). Studies have shown that plant products, including essential oils (EOs), extracts, and pure compounds, have significant effects on the resistant and biofilm-forming species of *Candida* (Ahmad et al., 2015). The natural compounds obtained from different parts of a plant, such as roots, leaves, and fruits, which have medicinal properties, show different medicinal effects once changes are applied to them (Rangel et al., 2018).

Recent studies have indicated that various natural compounds such as curcumin, cinnamaldehyde, eugenol, and thymol not only inhibit biofilm formation but also eliminate mature biofilm structures (Doke et al., 2014; Rangel et al., 2018). Additionally, the combination treatment with antifungal drugs and different natural compounds can be an effective solution to treat common diseases such as candidiasis due to its increased potency and effectiveness, lower drug toxicity, lower dosages, and reduced likelihood of developing resistant strains (Shinde et al., 2013).

Accordingly, this review concentrates on the interactions of different natural compounds with biofilm communities of *Candida* and various drug delivery platforms used for enhancing the therapeutic efficacy of natural compounds. The review also focuses on the synergistic antimicrobial activity of these compounds with antifungal drugs to facilitate their widespread use in clinical practice.

## Curcumin

Curcumin is one of the main phytochemical ingredients of *Curcuma longa* (Zingiberaceae family). Curcumin usually shows no to low cytotoxicity at active dose range and oral doses as high as 8–12 g per day. This compound has shown antioxidant, antiviral, antimalarial, anticancer, and antibiofilm characteristics in studies and show promising synergistic effects in combination with other drugs (Hewlings and Kalman, 2017; Shariati et al., 2019). Because of this, scientists are interested in employing this compound for degrading *Candida* biofilms. For instance, a recently published study reported that curcumin at high concentrations (0.1–2 mg/ml) effectively suppressed the planktonic and biofilm community of *Candida* (Narayanan et al., 2020). In this section, we have reviewed studies that have applied curcumin for inhibition and degradation of *Candida* biofilm in different stages.

As mentioned previously, fungal adhesion is an important stage in biofilm formation. Thus, if a compound disrupts microbial adhesion to different surfaces, it could be considered a potential antibiofilm agent. In this regard, Shahzad et al. (2014) reported that curcumin showed superior antibiofilm activity, remarkably suppressing initial *C. albicans* adhesion following pre-coating with this compound. This repressive effect diminished with the delay in curcumin exposure after the inoculation; however, curcumin could still suppress the biofilm formation by 50% when added 4 h after inoculation. The authors proposed that the antibiofilm activity of curcumin may be attributed to two α,β-unsaturated carbonyl groups that connect two phenol groups; additionally, the presence of two unsaturated double bonds in the central connecting chain could further boost its antibiofilm activity. Notably, curcumin reduced the expression of Agglutinin-like sequence 3 (*ALS3*), an adhesion-associated gene, and the hyphal protein HWP1 (Shahzad et al., 2014). The Als adhesive proteins are one of the most extensively studied virulence characteristics of *C. albicans*, and the elimination of *Als3* results in a significant reduction in fungal adhesion (Hoyer and Cota, 2016). These findings are in line with a recently published study that reported that curcumin (50 µg/ml) inhibited *C. albicans* adhesion, This activity of curcumin could be further boosted by the pre-treatment of yeast cells with the compound to obtain a remarkably greater inhibition of adhesion. Additionally, curcumin affected various immature morphological forms of *C. albicans*, such as germlings and yeast. Transcriptional analyses corroborated these findings and indicated that treatment with curcumin suppressed the expression of key adhesion-related genes *ALS3* and *ALS1*, while the expression of aggregation-related genes *AAF1* and *ALS5* increased (Alalwan et al., 2017). Notably, Shariati et al. (2019) reported that the human alveolar epithelial cells were well-spread, and there was no distinct change in morphology after 24 h of incubation with 50–200 µg/ml of curcumin compared to control cells. Therefore, curcumin could be used for the destruction of *Candida* biofilms without cytotoxic effects on human cells; however, more confirmatory studies are required in this area.

These data show that curcumin has an anti-adhesive activity against *C. albicans* and the compound interferes with the expression of adhesion- and biofilm-associated genes. It has also been reported that the treatment of *C. albicans* SC5314 strain biofilm with curcumin led to a decrease in the biofilm mass and proteinase and phospholipase activity of the biofilm community. However, proteinase gene expression was not downregulated after the curcumin treatment. *C. albicans* secretes proteinases and phospholipases for purposes such as host invasion, tissue degradation, and hypha formation. Thus, curcumin not only decreased the biomass of *C. albicans* biofilm but also suppressed critical pathogenic factors of this fungus (Chen et al., 2018).

The satisfactory performance of curcumin against *Candida* biofilms has led to the use of this compound for inhibiting drug-resistant strains. In this regard, Dong et al. (2021) used curcumin to restore the effectiveness of fluconazole against fluconazole-resistant *Candida*. Curcumin alone or in combination with fluconazole suppressed biofilm formation and yeast-to-hypha morphological transition in fluconazole-resistant *C. albicans*. Furthermore, this compound combined with fluconazole disrupted membrane permeability and reduced intracellular ATPase production. The synergism of curcumin and fluconazole has introduced this compound as a new therapeutic agent against drug-resistant *Candida* strains (Dong et al., 2021). However, there is limited data on the curcumin and antifungals interactions; thus, further studies are required.

In a biofilm community, microorganisms with different antibiotic resistance properties interact with each other; thus, treating an infection of a multi-species biofilm community is more difficult than that of a mono-species biofilm. In this regard, recent studies have evaluated the inhibitory effect of curcumin against mixed-species biofilms. In one such study, the authors assessed the inhibitory effect of curcumin against the dual-species biofilms formed by *Streptococcus mutans* and *C. albicans*. Curcumin suppressed *C. albicans* and *S. mutans* in both mono- and dual-species biofilms. Notably, *S. mutans* was more sensitive to treatment in the dual-species biofilms than in mono-species biofilms, while *C. albicans* was less sensitive to curcumin in dual-species biofilms. Curcumin downregulated the expression of quorum sensing-related genes and glucosyltransferase in *S. mutans* and *ALS1* and *ALS3* family in *C. albicans* in dual-species biofilms. Accordingly, curcumin, due to its inhibitory effect on various biofilm-associated molecular pathways of *C. albicans* and *S. mutans*, could be used for controlling caries-related dual-species plaque biofilms (Li et al., 2019).

In yet another investigation, curcumin exhibited antibiofilm activity against a mixed community of *Acinetobacter baumannii* and *C. albicans* (Raorane et al., 2019). In this mixed biofilm community, Hyr1p of *C. albicans* binds to the outer membrane protein of *A. baumannii*, FhaB; it has been reported that HYR1 knockdown remarkably decreased *C. albicans* hyphae binding to *A. baumannii* (Darwish Alipour Astaneh et al., 2017; Uppuluri et al., 2018). However, curcumin interactions and mentioned genes were not reported in this study (Raorane et al., 2019). Finally, the results of the study by Tan et al. (2019) showed that 2-aminobenzimidazole in combination with curcumin effectively degraded *C. albicans* and *Staphylococcus aureus* mixed biofilm on silicone surface. Collectively, curcumin showed good inhibitory effects against mixed bacterial and fungal biofilm. Hence, this compound could be used for mixed biofilm-associated infections such as oral disorders and infections of implants.

Nonetheless, like other natural compounds, curcumin has limited usage in the clinical setting due to its various drawbacks, including low oral bioavailability, water insolubility, rapid metabolism and degradation, poor absorption from the gut, and low blood plasma levels (Shariati et al., 2019). To address these issues, researchers have been interested in using various drug delivery platforms to enhance curcumin-antibiofilm efficacy. For instance, Rajasekar et al. (2021) used the curcumin-sophorolipid nanocomplex for the degradation of *C. albicans* biofilm. Sophorolipids, which are biologically-derived surfactants, are practical candidates for the delivery of hydrophobic molecules. The synthesized curcumin-sophorolipids remarkably suppressed fungal attachment, subsequent biofilm formation, filamentation, and maturation. Additionally, this compound downregulated different pathogenic genes such as *EFG1*, *ALS1*, *SAP8*, and *EAP1* (Rajasekar et al., 2021)*.* In another investigation, the authors used graphene oxide functionalized with curcumin and polyethylene glycol (PEG) for the eradication of *C. albicans* biofilm. The results showed that this compound decreased fungal proliferation, adhesion, and biofilm formation. Thus, the authors suggested that combining curcumin and PEG molecules on the graphene oxide surface results in a strong antibiofilm effect, which can also suppress the local growth of *C. albicans* cells not adhering to the surface (Palmieri et al., 2018). In line with these findings, the results of a recently published study demonstrated that curcumin-loaded chitosan nanoparticles (NPs) had acceptable antibiofilm effects against *S. aureus* and *C. albicans* mixed-species and mono-species biofilms. Microscopic analysis revealed that the synthesized compound reduced biofilm thickness and killed the microbial cells embedded in the biofilm on silicone surfaces. Thus, curcumin-loaded chitosan NPs could be used as an effective agent to eliminate infections caused by *Candida* biofilms, particularly those associated with mixed-species biofilms (Ma et al., 2020).

As is evident from the above studies, various drug delivery platforms could be used to enhance the antibiofilm efficacy of curcumin. However, data in this area are very limited, and more in-depth investigations should be carried out before the clinical usage of curcumin-based drug delivery platforms.

Finally, recent studies have employed the combination of photodynamic therapy and curcumin for *Candida* biofilm elimination. This type of treatment is in its initial stages, with the studies described in Table 1.

**TABLE 1:** Studies that have applied a combination of photodynamic therapy and curcumin for the elimination of *Candida* biofilm.

Year of publication (Reference)	Study model	*Candida* strain	Light source	Outcome
Quishida et al. (2016)	CUR-mediated API	*S. mutans*, *C. glabrata*, and *C. albicans* of different ages	LED light	Bothe biofilm communities (24 and 48 h) were inhibited by CUR-mediated API.
Merigo et al. (2019)	Combined use of CUR with blue diode laser and/or killer decapeptide	*C. albicans*	Laser	This combination showed an inhibitory effect against *C. albicans* biofilm community.
Ma et al. (2019)	CUR-mediated PDT	*C. albicans*	LED light	PDT significantly destroyed *C. albicans* biofilm; additionally, this treatment decreased biofilm-related and hypha-specific gene expression.
Kanpittaya et al. (2021)	Erythrosine/CUR Derivatives/Nano-Titanium Dioxide-Mediated PDT	*C. albicans*	Dental blue light in the 395–480 nm wavelength range	This combination was able to suppress *C. albicans* similarly to nystatin. All test photosensitizers demonstrated no toxicity on the fibroblast cells.
Jordão et al. (2020)	PDT mediated by two photosensitizing agents: CUR and Photodithazine^®^	*C. albicans*	LED (37.5 J/cm^2^ or 50J/cm^2^)	PDT mediated by LED-associated photosensitizing Photodithazine^®^ and CUR reduced the expression of genes associated with the attachment, biofilm development, and oxidative stress response.
Santezi et al. (2021)	PDT with various photosensitizing agents including CUR in DMSO, CUR-loaded microemulsion, CUR-loaded liquid crystal precursor system, and CUR-loaded chitosan hydrogel with a poloxamer	*C. albicans*	LED that provides a uniform emission from 440 to 460 nm, with maximum emission at 450 nm	CUR in DMSO was the only formulation able to remarkably decrease *Lactobacillus* and *C. albicans* biofilm cells viability.
Dovigo et al. (2011b)	CUR-mediated PDT	*C. albicans*	LED at different fluences	Decreased *C. albicans* viability in both planktonic and biofilm culture.
Hsieh et al. (2018)	CUR-mediated PDT was used in combination with fluconazole and as an independent therapy	*C. albicans*	Blue light	PDT significantly destroyed C. *albicans* biofilms; furthermore, PDT, in combination with fluconazole, suppressed *C. albicans* to a greater extent.
Andrade et al. (2013)	CUR-mediated PDT	*C. albicans*, *C. glabrata* and *C. dubliniensis*	LED	PDT decreased cell viability in the biofilm community.
Sanitá et al. (2018)	CUR-mediated PDT	*C. dubliniensis*	5.28 J/cm^2^ of LED light fluence	PDT reduced metabolism of biofilm-organized cells of *C. dubliniensis*.
Rocha et al. (2020)	PDT and curcumin microemulsion	*C. albicans*	LED at 430 nm	Decreased 3.497 log_10_ UFC/ml in *C. albicans* community.
Dovigo et al. (2011a)	CUR-mediated PDT	*C. albicans*, *C. tropicalis*, and *C. glabrata*	LED	Reduced metabolic activity of *Candida* biofilm community.

API, antimicrobial photodynamic inactivation; LED, light emitting diode; PDT, photodynamic therapy; CUR, curcumin; DMSO, dimethylsulfoxide.

## Thymol

Thymol, 2-isopropyl-5-methyl phenol, is an isomeric phenolic monoterpene with a strong odor and good solubility in alcohol and other organic solvents but is poorly water-soluble. Thymol is abundantly found in certain plants and has been used in traditional medicine for many years because of its different pharmacological properties, such as anti-rheumatic, antioxidant, antiseptic, anticancer, antiviral, antibacterial, and antifungal (Salehi et al., 2018; Schnitzler, 2019; Kowalczyk et al., 2020; Gholami-Ahangaran et al., 2022).

**FIGURE 1:**
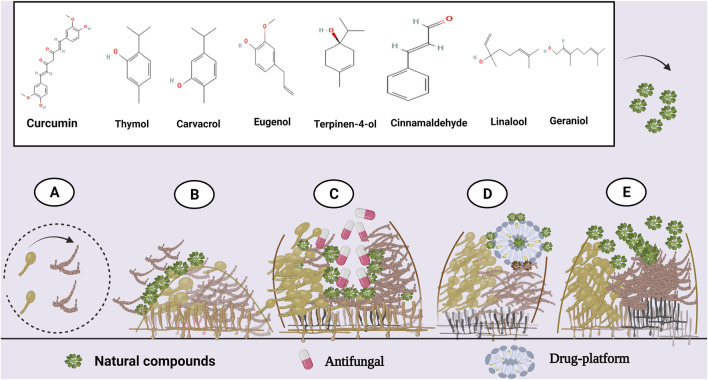
Antibiofilm activity of various natural compounds against *Candida*. **(A)** Inhibition of yeast-to-hypha morphological transition and **(B)** initial *Candida* adhesion. **(C)** Synergistic combination with antifungals. **(D)** Drug delivery platforms could enhance natural compounds' antibiofilm efficacy. **(E)** Destruction of an established biofilm. Created with BioRender.com

Scientists have evaluated the antibiofilm potential of this compound and reported that thymol could inhibit biofilm formation and has the capacity to reduce viable cells as well as eliminate mature biofilm community of various *Candida* (Figure 1) (Braga et al., 2008; Dalleau et al., 2008; De Vasconcelos et al., 2014; Shu et al., 2016; Chatrath et al., 2019). Further, gas chromatography-mass spectrometry analysis in recent studies has indicated thymol as the main ingredient of plant EOs, including that of *Carum copticum*, *Thymus vulgaris*, *Satureja hortensis* L, *Carum copticum* L, and *Trachyspermum ammi*. Notably, these EOs showed antibiofilm activity against *Candida* biofilms (Khan et al., 2014; Sharifzadeh et al., 2016; Arabi Monfared et al., 2020; Hejazi et al., 2021). Furthermore, *C. copticum* and *T. vulgaris* EOs could cause a >70% decrease in the hydrophobicity of the cell surface, and reduce hemolysin, and proteinase production (Khan et al., 2014).

Additionally, another study evaluated the antibiofilm effect of thymol against the mixed biofilm of *S. mutans* and *C. albicans*. Thymol significantly reduced the biofilm formation and virulence of both *S. mutans* and *C. albicans,* including acidogenicity (acid production from the dietary carbohydrates), acidurity (able to survive under lethal pH condition), and yeast-to-hyphal transition, in single- and mixed-species biofilm communities. Notably, these microorganisms did not show resistance against thymol, which could be associated with the fact that this compound showed regulatory effects on many genes/transcriptional regulators of both organisms (Priya et al., 2021b). The synergistic interaction between *S. mutans* and *C. albicans* within the carious biofilm leads to enhanced virulence of both pathogens. Recent investigations have demonstrated that the presence of *C. albicans* supports the extensive colonization of *S. mutans* in the dental biofilm (Waltimo et al., 2000; Priya et al., 2021b). Thus, thymol, due to its ability to inhibit fungal filamentation and morphogenesis, and impair *S. mutans* acidogenic and aciduric ability, could be a practical candidate for the prevention and treatment of early childhood caries; however, more confirmatory investigations are required.

Combined use of thymol with other natural compounds and antifungals also showed promising results in inhibiting the *Candida* biofilm community. Priya et al. (2021a) evaluated the synergistic effect of thymol and piperine, the major bioactive component of pepper, against biofilms formed by some clinical isolates of *C. albicans* through checkerboard assay. Both of these compounds at a concentration of 32 μg/ml inhibited the biofilm community of *C. albicans*, whereas the combined use of piperine and thymol indicated synergistic effects at four different combinations of concentrations (piperine and thymol at 8 and 8, 8 and 4, 8 and 2, and 4 and 8 μg/ml). The combination therapy led to the reduction of different virulence factors of *C. albicans*, including those related to the attachment, morphological transformation, and hyphal extension. In addition, hyphal elongation was controlled by limited exposure to synergistic combinations. The molecular assessment also confirmed these findings and showed that the expression of *tup1* and *nrg1*, the negative transcriptional regulators of filamentous growth, was upregulated under the influence of thymol and piperine. On the other hand, the expression of *ume6*, a positive regulator of the filamentation morphology, was remarkably downregulated. Collectively, these findings suggest that thymol and piperine could interact synergistically in exerting antihyphal and antibiofilm functions against *C. albicans* (Priya et al., 2021a). Along similar lines, another study evaluated the interaction of thymol and carvacrol against mono- and mixed-species growth of *Staphylococcus epidermidis* and *C. albicans*. This combination exhibited anti-adhesion, antibiofilm, and antihyphal actions. It also eliminated mono- and mixed-species highly tolerant persister cells and reduced the risk of resistance expansion (Swetha et al., 2020). Notably, a recently published study showed that the treatment of HEK 293 (human embryonic kidney) cells with 250 μg/ml thymol led to a loss of nearly 100% viability (Al-Ani and Heaselgrave, 2022). Therefore, it seems that the antibiofilm effect of thymol at a concentration of 32 μg/ml could be achieved without damaging the eukaryotic cells, although more studies are needed.

Another study reported synergistic effects of thymol and antifungal drugs against the drug-resistant (itraconazole and fluconazole) strains of *C. albicans* and *C. tropicalis*. Thymol reduced biofilm formation by these fungi either by preventing the attachment or interfering with the subsequent biofilm maturation. The microscopic evaluation also revealed deformed structures and disaggregation of *C. albicans* biofilm community cells and reduced hyphae formation of *C. tropicalis* biofilm cells at sub-minimum inhibitory concentrations (MICs) of thymol. Notably, thymol had a significant effect on the structure of the preformed biofilm of both *C. tropicalis* and *C. albicans*. Thymol also demonstrated synergy with fluconazole against both the planktonic and biofilm community of *C. tropicalis* and *C. albicans*. Nevertheless, synergy with amphotericin B (AMB) was evident only in the planktonic *Candida* cells (Jafri and Ahmad, 2020).

These results support the findings of Pemmaraju et al. (2013), who reported synergistic effects for thymol and fluconazole in the reduction of *C. albicans* biofilm formation. The adherence assay indicated 30% viability of fungal cells after 2 h of treatment with 0.05% (v/v) fluconazole and thymol. The authors proposed that improved antifungal action of fluconazole in the presence of thymol could be related to the alternations in the membrane permeability and fluidity and inhibition of ergosterol biosynthesis by thymol, which could lead to cytoplasm membrane disruption, cell contents leakage and cell wall degradation. The microscopic analysis confirmed this proposition and revealed alterations in the number of cells and structural design of *C. albicans* biofilm. Thymol with fluconazole showed the highest synergistic effect on biofilm inhibition compared to the other natural compounds, including eugenol and menthol (Pemmaraju et al., 2013).

Thus, synergistic combinations of thymol with other natural compounds such as piperine and carvacrol, as well as antifungals, could be a promising treatment for *C. albicans* biofilm-associated infections. Further, these combinations could possibly be used in the formulation of dentifrices that are particularly effective for oral candidiasis treatment. However, the mode of synergistic action of thymol with other natural compounds and antifungals is not clear at present; thus, these encouraging findings will need to be confirmed by *in vivo* and translational investigations before clinical usage can be realized.

## Carvacrol

Carvacrol, a phenolic monoterpenoid, is known as one of the main ingredients of EOs of various aromatic plants, such as oregano (*Origanum vulgare*), pepperwort (*Lepidium flavum*), and thyme (*Thymus vulgaris*). Carvacrol has been used as a food preservative, additive, and flavoring, as well as a fragrance in cosmetic products. Recent studies have revealed various biological activities of carvacrol, including antioxidant, anticancer, and antimicrobial activities (Sharifi-Rad et al., 2018; Javed et al., 2021). Because of its various properties, such as the presence of a free hydroxyl group and phenol moiety and hydrophobicity, carvacrol exhibited better antimicrobial activity than other volatile compounds (Sharifi-Rad et al., 2018). Various investigations have used carvacrol for inhibiting and degrading *Candida* biofilms (Dalleau et al., 2008).

Miranda-Cadena et al. (2021b) used various natural compounds, including carvacrol and cinnamaldehyde, against the planktonic and sessile cells of *Candida*. The authors evaluated the reduction in the biofilm metabolic activity and biomass during the adhesion as well as mature biofilm phases. Carvacrol remarkably reduced both the metabolic activity and biomass of the mature biofilm and even degraded the biofilms of azole-resistant strains. The microscopic observations also showed a loss of the cell viability of *Candida* biofilm community after carvacrol treatment. Cinnamaldehyde showed a better function in preventing biofilm formation, while carvacrol more effectively degraded mature *Candida* biofilm (Miranda-Cadena et al., 2021b). Thus, the combined use of carvacrol and cinnamaldehyde could be a promising approach for suppressing *Candida* biofilm-associated infections due to its interference with various biofilm formation stages. These results support the findings of Touil et al. (2020), who reported that using a combination of terpenoids (farnesol, carvacrol, and cuminaldehyde) could inhibit biofilm formation and kill drug-resistant *C. albicans*. Carvacrol significantly suppressed the biofilm formation of *C. albicans* (80% at 2,000 μg/ml). Additionally, carvacrol at a concentration of 2,000 μg/ml reduced the cells of *C. albicans* by more than 75% during the biofilm development with the co-isolated bacteria. Hence, carvacrol had destructive effects on the interactions between the mixed-species biofilms and suppressed the biofilm development. Notably, the combined use of carvacrol and farnesol revealed synergistic suppressive effects not only on *C. albicans* hyphae and yeast, but also on biofilms formed from single and mixed-species (Touil et al., 2020).

Gomes et al. (2022) reported that at a concentration of 100 μg/ml, carvacrol has a moderately toxic effect on L929 (mouse fibroblast) cells. Therefore, high concentrations of carvacrol can have a detrimental effect on eukaryotic cells, which should be evaluated in future studies.

In addition to cinnamaldehyde and farnesol, the combination of thymol and carvacrol also showed promising results for *C. albicans* biofilm inhibition. The carvacrol and thymol combination showed anti-adhesion, antibiofilm, and anti-hyphal activities. Further, this combination eliminated the highly tolerant persister cells of mono- and mixed-species of both *S. epidermidis* and *C. albicans* biofilms and was associated with less risk of resistance development (Swetha et al., 2020).

The combinations of carvacrol and antifungals also showed promising results for degrading *C. albicans* biofilm. Doke et al. (2014) reported that the mature biofilm of *C. albicans* was highly resistant to fluconazole but susceptible to natural compounds such as carvacrol. Sensitization of fungal cells by carvacrol at sub-inhibitory concentrations led to the inhibition of biofilm formation and destruction of mature biofilm of C. *albicans* at low fluconazole concentrations (0.032 mg/ml). On the other hand, thymol and eugenol combinations with fluconazole did not show useful interaction against C. *albicans* mature biofilms (Doke et al., 2014). Thus, carvacrol has the potential for treating drug-resistant *C. albicans*. It could additionally be used in combination with other natural compounds and antifungals for better antibiofilm action. Nonetheless, the efficacy of combination therapies with carvacrol and other natural compounds needs to be demonstrated in animal model studies and translational investigations before its use in clinical practice.

A recently published study reported carvacrol as the main ingredient of *Origanum majorana* L. EO. This EO exhibited antifungal and antibiofilm activities against *C. albicans*. In addition to biofilm inhibition, this EO also suppressed germ tube formation, one of the main virulence factors of *C. albicans*, and modulated cell surface hydrophobicity. Hence, carvacrol prevented biofilm formation, degraded mature *C. albicans* biofilm, and suppressed various virulence factors of this fungus. Accordingly, carvacrol has the potential to inhibit *C. albicans* pathogenesis and could be considered in patients with the risk of *C. albicans* infection or in the with common antifungals was without sufficient success (Kaskatepe et al., 2022). However, for this purpose, further *in vivo* and clinical studies are needed to evaluate the carvacrol efficiency in clinical setting and further enhance its activity against *Candida* biofilm.

To this end, various drug delivery platforms have been used for enhancing carvacrol antibiofilm efficacy. Vitali et al. (2021) used an ionic gelation procedure to synthesize chitosan NPs loaded with carvacrol. Afterward, the antibiofilm activity of carvacrol-loaded NPs was evaluated against *Candida* biofilms. The results showed that the antibiofilm activity of synthesized NPs was significantly dependent on the *Candida* species; accordingly, *C. krusei* and *C. tropicalis* showed the most susceptibility to the treatment. The authors suggested that the structures of biofilms in the studied species could affect the response to carvacrol-loaded NPs (Vitali et al., 2021). Studies indicate that the mature biofilm structure of *C. albicans* has a heterogeneous structure, composed of hyphae and blastospores surrounded by extracellular polysaccharides. On the other hand, *C. glabrata* biofilm community consists of yeast cells multilayer, intimately packed or arranged in cell clusters. Furthermore, *C. tropicalis* biofilm consists of a network of yeast, pseudohyphae, and hyphal yeast, with intense hyphal budding (Chandra et al., 2001; Bizerra et al., 2008; Silva et al., 2009; Vitali et al., 2021). Thus, it can be concluded that the antibiofilm effect of carvacrol and drug delivery platforms based on this compound should be evaluated against various *Candida* species. Since different *Candida* species have different biofilm structures, the antibiofilm efficacy of carvacrol could be different across various species.

In yet another investigation, the authors incorporated carvacrol into electrospun membranes of poly(lactic acid) (PLA) for the elimination of *C. albicans* and *S. aureus* mixed biofilm structure. Carvacrol showed good compatibility with PLA, improved the extensibility and flexibility of the matrix, and acted as a plasticizer. The gradual release of carvacrol from PLA membranes led to antimicrobial activity for up to 144 h and a reduction of biofilm formation by 88–95% for *C. albicans* and 92–96% for *S. aureus* in single and mixed cultures. Furthermore, the findings indicated a huge reduction in biomass and cell count, as well as metabolic activity of 24- and 48-h biofilms formed after the treatment (Scaffaro et al., 2018). These results highlight the potential of nanobiotechnology such as electrospun nanofibrous membranes as a practical delivery platform for carvacrol, an ecological substitution in developing new antibiofilm approaches, and a a promising agent for managing *Candida* biofilm-associated infections.

## Eugenol

Eugenol or 2-methoxy-4-[2-propenyl] phenol, a phenolic aromatic compound mainly derived from *Cinnamomum* and clove EO, is a natural microbiocidal compound belonging to the phenylpropanoid class of organic compounds and has been used for a long time as an analgesic in dentistry (Hassan et al., 2018; Wijesinghe et al., 2021). Eugenol can be synthesized by guaiacol allylation with allyl chloride or produced through a biotransformation process that involves microorganisms such as *Bacillus cereus*, *Corynebacterium* spp., and *Escherichia coli* (Abrahão et al., 2013). Eugenol also has anesthetic, neuroprotective, antidiabetic, insecticidal, analgesic, anti-inflammatory, and antifungal properties, which makes it a versatile natural ingredient that helps prevent and cure various disorders (Kozam, 1977; Reddy and Lokesh, 1994; Hassan et al., 2018; Nisar et al., 2021). Eugenol exhibits significant antifungal activity against various fungal species, including the dermatophytes *Aspergillus* and *Candida*, primarily due to damage to the fungal cell envelope and inhibitory action against biofilm communities and various virulence factors (Chami et al., 2005; Hassan et al., 2018). As a result, scientists are interested in using eugenol to suppress *Candida* biofilm communities and various cellular pathways of these fungi.

He et al. (2007) reported that eugenol reduced the adhesion capacity of cells and inhibited biofilm formation in *C. albicans*. Furthermore, the treatment of *C. albicans* cells with eugenol produced scant biofilms with suppressed filamentous growth (He et al., 2007). Notably, *C. albicans* is a dimorphic fungus that can switch from the yeast phase to the filamentous phase. The filamentous phenotype of this fungus is essential for pathogenicity and could have an important role in producing the spatially organized architecture seen in mature, highly structured *C. albicans* biofilms (López-Ribot, 2005). These data suggest that eugenol inhibited biofilm formation in *C. albicans*, affected the fungal cells’ morphogenesis, and weakened their invasive capacity (He et al., 2007).

Furthermore, molecular docking analysis indicated that eugenol impacts the *C. albicans* Als3 protein (El-Baz et al., 2021). The binding capacity of eugenol to Als3 was higher than that of other natural compounds such as cinnamaldehyde (Hoyer and Cota, 2016). Thus, the interaction of eugenol and Als3 may be the underlying mechanism of inhibition of *C. albicans* adhesion and biofilm formation by this compound (El-Baz et al., 2021).

Additionally, as a significant constituent of *Cinnamomum*, eugenol has demonstrated promising antibiofilm effects against *Candida*. In a study conducted in 2018, the authors used the *Cinnamomum zeylanicum* EO to inhibit *Candida* biofilm. The results showed that *C. zeylanicum* EO (500 μg/ml) significantly inhibited the formation of *Candida* biofilm and reduced mono- (*C. tropicalis*) and multi-species biofilm communities formed by these fungi. On the other hand, this EO at 1,000 μg/ml concentrations did not reduce human red blood cell viability. Notably, phytochemical evaluations pointed out eugenol as the main component (68.96%) of the EO extracted from *C. zeylanicum* Blume leaves (Rangel et al., 2018). A recent study also reported eugenol as the main compound (77.22%) of *Cinnamomum verum* EO. This EO suppressed initial adhesion, germ tube formation, and biofilm progression of *Candida*. Furthermore, the microscopic assessments showed decreased hyphal formation, cell wall damage, and cell shrinkage after treatment with this EO. Nevertheless, no lethal effect of *C. verum* EO was observed using the *Galleria mellonella* experimental model at the various concentrations tested (Wijesinghe et al., 2020).

These observations support the findings by Wijesinghe et al. (2021), who reported eugenol as the main compound of *C. verum* EO. This EO also suppressed established biofilm and hyphal production in *Candida* by causing cell wall injuries and cellular shrinkage. On the other hand, *C. verum* EO did not exhibit any effect on HaCaT (aneuploid immortal keratinocyte cell line from adult human skin) cells (Wijesinghe et al., 2021). Hence, *Cinnamomum* EO contains eugenol as its major chemical component, which can suppress the biofilm community and cause cell wall damage in *Candia*. Various studies indicate low cytotoxicity of this compound in different human cell lines.

Eugenol can be used to treat *Candida* biofilm-associated oral disorders. In a study conducted by Jafri et al. (2019), eugenol was used to inhibit single and mixed biofilms of *S. mutans* and *C. albicans* resistant to AMB, itraconazole, and ketoconazole. The interaction of these two significant oral pathogens may result in recalcitrant and resistant infections in the oral cavity, thereby increasing the complexity of managing them. Microscopic analysis revealed that eugenol treatment leads to cell shape alterations, reduced cell aggregation, and disordered single and mixed biofilms. At sub-MIC of 100 μg/ml, eugenol significantly suppressed single and mixed biofilms formed by the drug-resistant strains of the two oral pathogens (Jafri et al., 2019). Another investigation also reported inhibitory effects of eugenol against the planktonic and biofilm community of *C. tropicalis* and *Candida dubliniensis* (dose-dependent and fluconazole-resistant strains) isolated from the oral cavity of HIV-positive patients. No metabolic activity was detected in the biofilms after 24 h of treatment with eugenol (500 μg/ml); moreover, this compound markedly decreased the biofilm cells on denture material surfaces. It is noteworthy that eugenol significantly inhibited the adhesion of *Candida* to the polystyrene and HEp-2 cells; thus, the authors proposed that eugenol may have an additional beneficial effect in the treatment of local candidiasis (De Paula et al., 2014).

Previous studies have investigated the combination therapy of eugenol and fluconazole against *Candida* biofilms. A study conducted in 2014 described a synergistic effect of fluconazole and eugenol against a planktonic community of *C. albicans*. The established biofilm of this fungus was highly resistant to fluconazole, while sensitization of fungal cells by eugenol (at sub-inhibitory concentrations) led to the inhibition of biofilm formation at low fluconazole concentrations. The authors hypothesized that eugenol destabilizes the cytoplasmic membrane and causes specific signal intervention. Hence, this compound caused sensitization of *C. albicans* biofilms, boosting fluconazole penetration and leading to the inhibition of biofilm formation (Doke et al., 2014).

Additionally, Khan et al. reported that preformed *C. albicans* biofilms showed ≥1,024 x increased resistance to fluconazole but no increased tolerance to eugenol. This compound inhibited the biofilm formation in *C. albicans* and exhibited a synergistic interaction with fluconazole against biofilms formed by the test strains. The microscopic evaluation showed the effect of eugenol on the cell membrane integrity, as evidenced by the shrinkage of the cell surface in the biofilm cells. Therefore, the authors proposed that when eugenol, which possesses cidal activity against the biofilm cells, is combined with fluconazole, the drug’s fungistatic nature is converted to fungicidal (Khan and Ahmad, 2012).

In line with these observations, another investigation performed in 2020 in India reported that eugenol suppressed *S. mutans* and *C. albicans* mixed biofilms. The sessile MIC of fluconazole was increased up to 1,000-fold over planktonic MIC. Notably, eugenol was highly synergistic with fluconazole against *C. albicans* single and mixed biofilms. The microscopic analysis also confirmed these findings and showed distorted cell structure, decreased matrix production, and elimination of single and mixed biofilm cells of *S. mutans* and *C. albicans* in the samples treated with eugenol. Therefore, eugenol may disrupt cell membrane integrity and boost the drug entry into the microbial cell. This phenomenon increases the antimicrobial drug availability in the deeper layers of biofilm, consequently improving treatment efficacy (Jafri et al., 2020).

The results reported in the above studies indicate that besides having fungicidal activity, eugenol could potentially inhibit the adhesion capacity of planktonic cells of *Candida*. The compound suppressed biofilm formation and destroyed the established biofilm of these fungi formed on various surfaces. Thus, eugenol is a natural product with potential for non-toxic therapeutic application in the treatment of candidiasis by interfering with the most important virulence factors of *Candida* species, such as germ tube formation, adhesion to various host surfaces, and biofilm formation. However, the exact mechanism of antifungal and antibiofilm effects of eugenol against *Candida* is not clearly understood; hence, further studies are required.

## Terpinen-4-ol

Terpinen-4-ol is a monoterpene and the main bioactive ingredient of tea tree oil (TTO). It is found in many aromatic plants such as mandarins, Japanese cedar, black pepper, oregano, oranges, and New Zealand lemonwood tree (Pino et al., 2003; Shapira et al., 2016). Monoterpenes are major plant-derived secondary metabolites extensively found in natural products, such as vegetables, herbs, and fruits. They are known to be related with the plant defense activity (Gershenzon and Dudareva, 2007; Shapira et al., 2016). Previous studies have reported antibacterial and antifungal properties of terpinen-4-ol (Dryden et al., 2004; Mertas et al., 2015). Thus, scientists have been interested in applying this compound for the inhibition and degradation of *Candida* biofilm.

Francisconi et al. (2020a) reported that TTO and its main component terpinen-4-ol suppressed the growth of *C. albicans*. The application of TTO and terpinen-4-ol for 60 s (rinse simulation) at the concentrations of 17.92 mg/ml and 8.86 mg/ml, respectively, disrupted the biofilm formation of *C. albicans*. Thus, terpinen-4-ol could suppress biofilm formation at a lower concentration than the TTO. Furthermore, the microscopic analysis showed that this compound penetrated the cytoplasmic membrane, caused *C. albicans* cell membrane disruption, and interfered with the pathogen cell’s integrity and physiology. The authors suggested that the lipophilicity of TTO and terpinen-4-ol could be related to the mentioned antifungal characteristics (Francisconi et al., 2020a). In another study, the authors used TTO and terpinen-4-ol for inhibiting the biofilm community of *C. albicans*. TTO and terpinen-4-ol killed the planktonic *C. albicans* isolates; also, terpinen-4-ol showed potent activity against the biofilm structure of this fungus. Notably, terpinen-4-ol was not cytotoxic at 0.5 × MIC50 (the concentration able to suppress *C. albicans* growth) against OKF6-TERT2 epithelial cells. Accordingly, the authors concluded that the use of terpinen-4-ol, the main ingredient of TTO, has advantages over the complete EO in terms of product consistency as well as safety and may be useful for preventing and treating various *C. albicans* biofilm-associated infections, especially established oropharyngeal candidiasis (Ramage et al., 2012).

Another study has shown that the combined use of the terpinen-4-ol and antifungals like nystatin exhibited synergistic effects in inhibiting *Candida* biofilms. Nystatin binds to the steroids within the cell membranes of susceptible fungi, interferes with the cell membrane permeability, and causes the cytoplasmic content to leak from the cell. Nonetheless, this antifungal drug has some adverse effects in humans, such as an unpleasant taste and gastrointestinal disorders, and fungal resistance to nystatin has been reported (Francisconi et al., 2020a). Hence, the synergistic effects of nystatin with various natural compounds could help reduce the nystatin dosage and its unwanted side effects, prevent the development of resistance to this antifungal drug and boost its clinical application.

In a recent study, the authors assessed the antifungal activity of terpinen-4-ol in combination with nystatin against *C. albicans* and *C. tropicalis* mixed-species biofilm. The results indicated that terpinen-4-ol and nystatin at 4.53 mg/ml and 0.008 mg/ml, respectively, were able to suppress biofilm growth. A synergistic antifungal effect was observed with the drug combination, and a reduction of nystatin inhibitory concentration up to eight times in the mono-species biofilm of *C. albicans*, and up to two times in mixed-species biofilm was achieved. Further, terpinen-4-ol limited the adhesion of *C. tropicalis* to oral keratinocytes, while this compound did not show inhibitory effects on the fungal enzymatic activity (Tonon et al., 2018). Terpinen-4-ol, like other monoterpenes, has a hydrophobic nature and a capacity for binding to different lipophilic structures of fungi, such as the plasma membrane. Accordingly, this compound enhanced non-specific cell permeability and caused the loss of essential electrolytes required for cell survival (Carson et al., 2006). Thus, it seems that terpinen-4-ol disturbs the *Candida* cell membrane permeability and increases the penetration of nystatin to the deeper layers of biofilms.

In another interesting study, Francisconi et al. (2020b) developed a liquid crystalline system (LCS) incorporated with nystatin and terpinen-4-ol to assess its antibiofilm and synergistic/modulatory function against *C. albicans*. The LCS containing nystatin and terpinen-4-ol significantly suppressed the *C. albicans* growth and biofilm formation at a lower concentration of each compound, displaying a synergistic action. The mucoadhesion analyses indicated that the addition of artificial saliva to the LCS improved its mucoadhesive and viscosity properties. Thus, the authors suggested LCS incorporated with nystatin and terpinen-4-ol as a promising antibiofilm agent for preventing and treating the *C. albicans* infection; however, more confirmatory studies are required (Francisconi et al., 2020b). Thus, nystatin combination with terpinen-4-ol could lower the nystatin quantity used in antifungal treatments, thereby diminishing unwanted side effects.

In addition to the above drug platform for terpinen-4-ol delivery, the researchers have developed other platforms for boosting the antibiofilm efficacy of this compound against *Candida* biofilms and overcoming high volatilization and nonwettability characteristics of terpinen-4-ol, which limits its clinical usage,. In one of such studies, the authors used PEG-stabilized lipid NPs loaded with terpinen-4-ol for *C. albicans* biofilm elimination. The results showed that this platform could suppress the biofilm community of this fungus. Various analyses confirmed that terpinen-4-ol-loaded lipid NP’s antibiofilm activity results from its capacity to destroy the cell membrane structure of *C. albicans* and block the respiratory chain by inhibiting succinate dehydrogenase attached to the inner mitochondrial membrane of cells (Sun et al., 2012). In line with these results, Souza et al. (2017) also used nanostructured lipid carriers (synthesized by a proprietary method) for the delivery of TTO and degradation of *Candida* biofilms. The chromatographic profile indicated that the TTO oil was in accordance with ISO 4730, with 41.9% terpinen-4-ol as a major constituent. The synthesized TTO NPs significantly reduced the biofilm formation of various *Candida* species including *C. albicans*, *C. glabrata*, C. *parapisilosis*, *C. tropicalis*, and *Candida membranafaciens.* Furthermore, the TTO NPs lowered the levels of the biofilm protein and exopolysaccharides (Souza et al., 2017).

The above studies suggest that the use of nanobiotechnology for delivery of terpinen-4-ol could enhance its antibiofilm activity, making it a suitable alternative against *Candida* biofilm-associated infections; however, there are very limited data in this area, and more studies are required. Collectively, terpinen-4-ol could be used for the treatment of *Candida* infections, especially for use in prophylactic oral hygiene products such as mouth rinses and denture cleansers.

## Cinnamaldehyde

Recent investigations have focused on the cinnamaldehyde’s ability to inhibit *Candida* biofilm formation, thereby limiting the extension of decreasing or resistant antifungal selective pressure. Cinnamaldehyde, one of the main *Cinnamomum* ingredients constituting about 65% of it, could have an antimicrobial activity due to its acrolein group (α,β-unsaturated carbonyl moiety)*.* Cinnamaldehyde does not suffer from antibiotic resistance despite its strong effect on pathogenic infections (Bae et al., 1992). In recent years, in addition to its antimicrobial effect, scientists have been interested in using cinnamon and its derivative components, especially cinnamaldehyde, to inhibit microbial biofilms (Kosari et al., 2020). This section primarily focuses on the role of cinnamaldehyde in the suppression and elimination of *Candida* biofilms to facilitate its possible widespread use in clinical practice.

Choonharuangdej et al. (2021) used Cinnamomum oil to inhibit preformed *C. albicans* biofilm on dental devices made from heat Polymerized Polymethyl Methacrylate (PMMA) resin. PMMA is associated with the development of mild to severe candidiasis in patients who wear it. Cinnamomum oil eliminated 99% of the pre-established *Candida* biofilm on this resin. Additionally, coating the PMMA samples with this oil for 24 h also reduced the formation of *C. albicans* biofilm by almost 70.0% (Choonharuangdej et al., 2021). In addition, the results of a recently published study illustrated intense antifungal activity for cinnamaldehyde against *Candida* species isolated from patients with oral candidiasis. Moreover, this compound decreased the metabolic activity and biomass of mature biofilms (Miranda-Cadena et al., 2021a). Notably, the reduction of biofilm biomass may have an essential role in handling resistant infections because biofilms are a source for dispersal of cells, resulting in the formation of new biofilms and enhancement of virulence and adhesion (Uppuluri et al., 2010; Nobile and Johnson, 2015).

It should be noted that the results of a study published in 2021 showed that *Cinnamomum verum* EO at concentrations of 1,000–2,000 µg/ml has no toxic effects on normal human keratinocyte cell line (Wijesinghe et al., 2021).

In addition to the research mentioned above, some studies examined the molecular interactions of cinnamaldehyde with *Candida* biofilms and obtained interesting results. A study conducted by El-Baz et al. (2021) reported that *C. verum* EO had an inhibitory effect against the biofilms formed by *C. albicans* strains isolated from different clinical samples. This EO also suppressed the hemolysin and phospholipase activity of this fungus. Microscopic images showed a decreased biofilm formation due to the suppressed adhesion. Molecular docking showed that cinnamaldehyde, as the main component of *C. verum* EO, has an impact on Als3 (El-Baz et al., 2021). Thus, the interaction of cinnamaldehyde and Als3 holds promise for using this compound to inhibit *C. albicans* adhesion and biofilm formation (El-Baz et al., 2021).

Another study discovered that cinnamaldehyde inhibited biofilm formation and affected *Candida* cellular development, as detected by specific features such as the absence of chlamydoconidia and the expression of rare pseudo-hyphae. Molecular docking analysis revealed that cinnamaldehyde presented negative ligand-receptor interaction energy at the studied targets with the most affinity for squalene epoxidase and thymidylate synthase. Therefore, the authors hypothesized that cinnamaldehyde could restrict the formation of biofilms in *Candida* by affecting important targets present in the fungal cell and nucleus; however, further docking studies are required for the precise identification of the proteins targeted by cinnamaldehyde (Da Nóbrega Alves et al., 2020). Furthermore, *Gupta* et al. discovered that cinnamaldehyde destroyed the biofilms formed by *C. glabrata* clinical isolates on biomaterials such as urinary catheters and contact lenses. Cinnamaldehyde enhanced cell lysis, ROS production, and plasma membrane ergosterol content; It also suppressed the phospholipase, catalase, and proteinase activity of *C. glabrata* cells. Detailed molecular analysis revealed that cinnamaldehyde downregulated the expression of *FKS1*, *AUS1*, *KRE1*, and *CDR1* genes related to the 1,3-β-glucan synthase, sterol importer, GPI-anchored protein, and multi-drug transporter, respectively. As a result, the authors hypothesized that interactions between cinnamaldehyde and ergosterol, result in the formation of pores that alter the permeability and integrity of the cell membrane and ultimately result in intracellular content leakage and cell lysis (Gupta et al., 2018). According to the above-mentioned studies, the interaction of cinnamaldehyde with different cellular pathways of *Candida* could inhibit biofilm formation and suppress various virulence phenotypes of this fungus. However, the data on this subject are scarce, necessitating additional research.

Due to the poor solubility of cinnamaldehyde in aqueous solutions and its instability and volatility, the typical dosage of oral rinses enables only short exposure to the compound and may not be suitable to treat active infections. Different drug platforms have been considered in recent years to overcome this limitation and increase the efficiency of cinnamaldehyde against *Candida* biofilms. Mishra et al. (2021a) addressed the above issue by incorporating cinnamaldehyde into electrospun gellan (GA)/polyvinyl alcohol (PVA) nanofibers. These nanofibers eliminated 50.45% and 89.29% of *C. albicans* and *C. glabrata* biofilm, respectively. The authors proposed that the unique characteristics of *C. albican’s* biofilm, such as a multi-layered structure consisting of blastosphores, hyphae, and pseudohyphae, could be related to the lower antibiofilm activity of the nanofibers against it. On the other hand, *C. glabrata* biofilm, consisting of a monolayer of cells with psuedohyphal and yeast morphology and a discontinuous matrix is more susceptible to the treatment (Mishra et al., 2021a). Another investigation evaluated the antibiofilm effect of cinnamaldehyde encapsulated in multilamellar liposomes (CM-ML) against *C. albicans* clinical isolates. CM-ML containing cinnamaldehyde showed improved fungicidal effects than cinnamaldehyde alone, possibly due to sustained release of cinnamaldehyde in the encapsulated form (Khan et al., 2017).

Finally, in a recent study, cinnamaldehyde was loaded onto poly (DL-lactide-co-glycolide) (PLGA) NPs (CA-PLGA NPs) and their antibiofilm activity against *C. albicans* was assessed. The results showed that the amount of active ingredient loaded in CA-PLGA NPs was much lower than the free cinnamaldehyde, and a strong antifungal effect was obtained even at this rate. Notably, the post-biofilm application of CA-PLGA NPs was more effective than the pre-biofilm application (Gursu et al., 2021). Therefore, the use of drug delivery systems for increasing the effectiveness of cinnamaldehyde may allow the use of lower doses to eliminate *Candida* biofilms. Different drug platforms can also mitigate the cytotoxicity caused by cinnamaldehyde. In this regard, future studies should focus more on encapsulating cinnamaldehyde in different carriers to enhance *Candida* biofilm inhibition.

## Linalool

Linalool, an unsaturated monoterpene alcohol with a pleasant odor, is produced by over 200 plant species belonging to different families, such as Rutaceae (citrus), Lauraceae (cinnamon, laurels, rosewood), and Lamiaceae (mints) (Kamatou and Viljoen, 2008; Elsharif et al., 2015). Linalool is known as a chemical intermediate in the biosynthesis of vitamin E. Additionally, this compound is one of the main ingredients of various EOs with different biological activities such as antiplasmodial, anti-inflammatory, antinociceptive, antihyperalgesic, and antibacterial (Peana et al., 2006; Van Zyl et al., 2006; Kamatou and Viljoen, 2008). Since linalool’s discovery, this compound has been extensively used in various clinical and industrial investigations. Previous studies have introduced linalool as a promising antibiofilm agent against *Candida* biofilm, and this section reviews these studies.

In one of these studies, the authors reported linalool as the main component (29%) of Iranian *S. macrosiphon* EO. This EO at concentrations of 4–8 μg/ml showed antibiofilm activity against various species of *Candida* (Motamedi et al., 2019). Another study also showed that linalool-rich EO from *Croton cajucara* inhibited the growth of various oral microorganisms such as *C. albicans*. In this study, purified linalool also inhibited the *C. albicans* biofilm community. The electron microscopy indicated that linalool treatment led to abnormal germination and cell size reduction in the fungus. Germ tube production is an essential virulence factor for *C. albicans* invasion; the inhibition of this virulence factor could be a possible treatment option for *Candida* infections (Alviano et al., 2005). Souza et al. (2016) evaluated the cytotoxicity effects of linalool on normal human lung fibroblasts, and the compound’s IC50 value (50% inhibition of cell line) was 1,253.00 ± 2.83 μg/ml.

Other studies evaluated the cellular interactions of linalool and *Candida* biofilms. A recent study indicated that linalool significantly suppressed *C. albicans* biofilm formation without affecting the planktonic cell growth. This compound, combined with α-longipinene (the main ingredient of cascarilla bark oil) and helichrysum oil, synergistically reduced biofilm formation. Further, linalool potentially inhibited *C. albicans* hypha formation. Notably, the biofilm community *C. albicans* is composed of hyphal, psuedohyphal, and yeast elements, and the transition from the yeast-to-hyphal stage could be an important virulence factor (Manoharan et al., 2017a). Abnormal biofilm communities developed in strains with defects in hyphae formation; the biofilm structure was thin, densely packed with yeast cells, and easily detached from the substratum (Nobile and Mitchell, 2006). Accordingly, the authors proposed that linalool-mediated inhibition of *C. albicans* biofilm formation could result from its interference with the production and induction of filaments. Additionally, using linalool with other natural compounds as a combinational therapy could be more useful in fungal biofilm elimination (Manoharan et al., 2017a). These results support the findings of Hsu et al. (2013), who reported that linalool eliminated the preformed biofilm structure of *C. albicans* by >80% at 2 × MIC. Further, this compound suppressed germ tube and biofilm formation in this fungus. The molecular analysis indicated that linalool downregulated the expression of various pathogenic genes in *C. albicans* such as *ALS3* and *HWP1* (adhesion-related genes), *CPH1* and *CYR1* (encoding components of the Mitogen-activated protein kinase and CAMP-Protein kinase A hyphal formation regulatory pathways, respectively) as well as *EED1*, *HGC1*, and *UME6* (long-term hyphae maintenance-associated genes) (Hsu et al., 2013).

These findings indicate that linalool not only suppresses *C. albicans* attachment and biofilm formation but also exerts an inhibitory effect on the pathways that regulate hyphal transformation and the mechanisms related to hyphal maintenance. The above studies highlight the antibiofilm activities of linalool for treating *Candida* biofilm-associated infections, such as those associated with implant medical devices, and the potential application of this compound in treating human and animal infections. However, more studies are required for a better understanding of linalool’s interactions with *Candida* biofilms.

## Geraniol

Geraniol, trans-3,7-dimethyl-2,6-octadien-1-ol, is an acyclic monoterpene alcohol detected in various EOs such as those of geranium, rose, and lemongrass. Because of its distinctive floral aroma and sweetness, this compound is one of the main commercial fragrance compounds that has been used in 58% of perfumes, 26% of cosmetic creams, and 40% of deodorants (Chacón et al., 2019). Recent studies have suggested additional commercial uses for geraniol as an anti-inflammatory, pain-relieving, and anticancer compound (Peralta-Yahya and Keasling, 2010; De Cássia Da Silveira E Sá et al., 2013; Chacón et al., 2019). The antimicrobial activity of this compound against the planktonic communities of various *Candida* species has also been reported. Thus, researchers have been interested in employing geraniol for inhibiting and degrading the biofilm structures of these fungi (Leite et al., 2015; Gupta et al., 2021a).

A recent study reported that geraniol not only suppressed both planktonic and sessile growth of various *C. glabrata* strains, but also degraded the mature biofilm structure of these fungi. Microscopic and molecular analysis indicated that geraniol lowered the eDNA and carbohydrate content and hydrolytic enzyme activity in *C. glabrata* extracellular matrix. This compound targeted β-glucan and chitin in the cell wall, reduced the cell membrane ergosterol content, altered the activity of mitochondria, increased Ca^2+^ uptake, and blocked ABC drug efflux pumps. Furthermore, the real-time polymerase chain reaction analysis indicated that the expression of *ERG4* and *CDR1*, which is related to ergosterol biosynthesis and multi-drug efflux transport, respectively, was downregulated by geraniol (Gupta et al., 2021a). These results align with the results obtained by Singh et al. (2016), who evaluated the interactions of geraniol with *C. albicans* cellular pathways. The findings revealed that geraniol suppressed both the biofilm formation and virulence attributes of hyphal morphogenesis in *C. albicans*. Further, this compound caused depletion of ergosterol levels, alteration of plasma membrane ATPase activity, iron genotoxicity, homeostasis dysregulation, and mitochondrial dysfunction (Singh et al., 2016).

**FIGURE 2:**
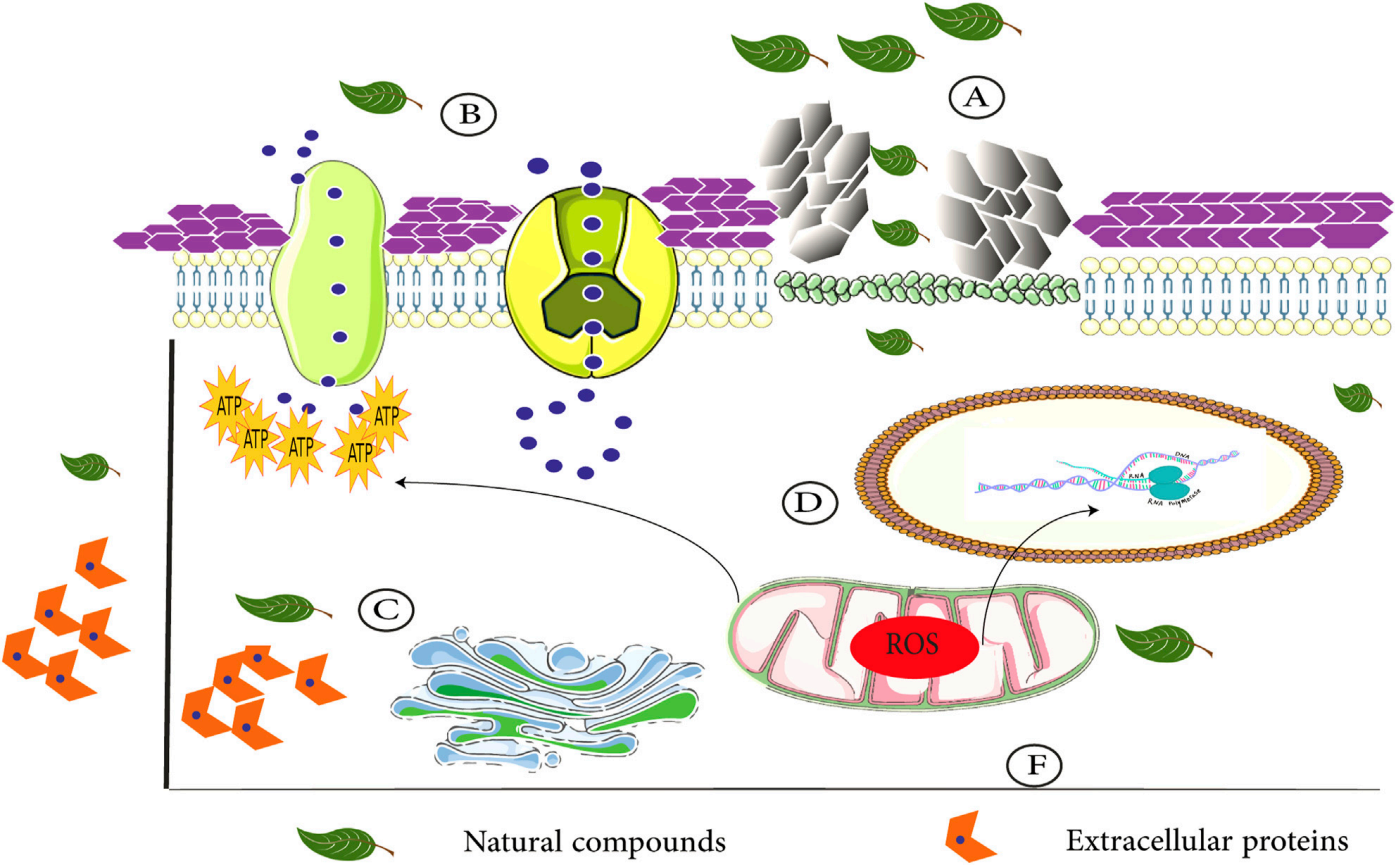
Different interactions of natural compounds with *Candida* cellular pathways. **(A)** Ergosterol biosynthesis inhibition (cytoplasm membrane disruption) and cell wall degradation. **(B)** Negative effects on various *Candida* cellular pathways such as ABC drug transport: additionally, modulated efflux pump activity. **(C)** Blockage of proteinase and phospholipase activity and intracellular ATPase production. **(D)** Downregulation of the expression of various adhesion and biofilm-associated genes. **(F)** Natural compounds lead to mitochondrial dysfunction.

In addition to the above *Candida* species, geraniol (at 500 μg/ml) remarkably reduced the number of viable biofilm cells and inhibited biofilm formation in a clinical strain of *C. tropicalis* after exposure for 48 h. The microscopic examination after treatment with this compound revealed a complex architecture with the upper layer of filamentous cells and basal layer of yeast cells. Additionally, geraniol at concentrations that were active against *C. tropicalis*, did not show cytotoxic effects on normal human lung fibroblasts (Souza et al., 2016). The above findings indicate that geraniol has negative effects on various *Candida* cellular pathways such as ABC transporter-mediated drug transport, ergosterol, chitin, and glucan biosynthesis and on cell cycle progression and mitochondrial activity (Figure 2). Thus, geraniol should be considered as a new antifungal agent against various *Candida* species infections.

The combination of geraniol and fluconazole also showed promising outcomes for the inhibition of *C. albicans* biofilm. The results of a study published in 2018 demonstrated that geraniol decreased biofilm biomass, impaired fungal adherence to the epithelial cells, and was synergistic with fluconazole against *C. albicans*. The authors proposed that geraniol modulated efflux pump activity by binding to the active site of CaCdr1p (drug efflux pump). Hence, modulation of efflux pump activity by geraniol and fluconazole synergism represents a promising approach for combinatorial treatment of candidiasis (Singh et al., 2018). Geraniol (at a MIC of 152 μg/ml) showed a good inhibitory effect against both fluconazole-sensitive and resistant *C. albicans* strains. The use of fluconazole with geraniol boosted the antifungal function of the combination, mainly against the resistant strain. Geraniol caused cell wall thickening and irregularities in the membrane structure, reduced ergosterol biosynthesis, and inhibited biofilm formation in *C. albicans* (Cardoso et al., 2016). Collectively, geraniol could boost the fluconazole activity by inhibiting *C. albicans* efflux pump activity and degrading the external structure of this fungi. The ability of geraniol to sensitize *Candida* cells to fluconazole opens up new options for combination therapy; however, more studies in animal models and clinical trials are needed before clinical application of this combination becomes a reality. Its noteworthy that other studies have used natural compounds for inhibition and destruction of *Candida* biofilm are presented in Table 2.

**TABLE 2:** The studies that have employed various natural compounds for inhibition and degradation of the *Candida* biofilm. Natural compounds that are the main ingredient of their-associated plants are reported here.

Year of publication (Reference)	Natural compound	Source of natural compound	*Candida* species (other microorganisms)	Outcome
Quatrin et al. (2017)	1-8-Cineol	*Eucalyptus globulus* oil	*C. tropicalis* and *C. glabrata* (Pseudomonas aeruginosa)	Nanoemulsion containing *eucalyptus globulus* oil was more efficient in the destruction of biofilm when compared to free oil.
Alves et al. (2019)	Borneol	*Thymus carnosus* EO	*C. albicans*	Disrupted preformed biofilm.
Manoharan et al. (2017b)	Borneol	Purchased directly from Sigma-Aldrich	*C. albicans*	Reduced biofilm formation.
Alfaifi et al. (2020)	Caffeine	NR	*C. albicans*	Caffeine at 32.00 and 16.00 mg/ml remarkably reduced the metabolic activity of *C. albicans* biofilm.
Alves et al. (2019)	Camphene	*Thymus carnosus* EO	*C. albicans*	Disrupted preformed biofilm.
Ivanov et al. (2021)	Camphor	Purchased directly from Sigma-Aldrich	*C. parapsilosis*, *C. albicans*, *C. glabrata* and *C. krusei*	Reduced established biofilm and hyphal formation.
Manoharan et al. (2017b)	Camphor	Purchased directly from Sigma-Aldrich	*C. albicans*	Significantly decreased biofilm community and hyphal formation. Additionally, downregulated some biofilm-related and hypha-specific genes.
Santos et al. (2017)	Camphor	NR	*C. albicans*	Diamond-like carbon films were incorporated with camphor, reducing the biofilm formation of 99% of *C. albicans.*
Yang et al. (2021)	Carnosol	NR	*C. albicans*	Inhibited biofilm formation and development.
Baygar et al. (2018)	Carvacrol	Purchased directly from Sigma-Aldrich	*C. albicans* (various bacterial strains)	Incorporation of carvacrol into the soft liner reduced C. *albicans* biofilm formation.
Pazarci et al. (2019)	Carvacrol	*Mentha longifolia* EO	*C. albicans* (various bacterial strains)	*C. albicans* was susceptible to EO
Chatrath et al. (2022)	Citral	Purchased directly from Sigma-Aldrich	*C. tropicalis*	Citral had various effects on biofilm-associated proteins.
Garcia et al. (2021)	Citral	Lemongrass EO and geranium EO	*C. albicans*	Chitosan microparticles loaded with EO showed an inhibitory effect against biofilm.
Gao et al. (2020)	Citral	Lemongrass (*Cymbopogon flexuosus*)	*C. albicans* and *C. tropicalis* (*S. aureus*)	Decreased cell viability and biofilm biomass of each species in the biofilm. Furthermore, citral downregulated virulence factor and hyphal adhesins in *C. albicans*.
Chatrath et al. (2019)	Citral and Thymol	Purchased directly from Sigma-Aldrich	*C. tropicalis*	These compounds indicated inhibitory effects against the planktonic and biofilm community. Citral and thymol targeted cell membrane and cell wall, respectively, and had an inhibitory effect on cell membrane biosynthesis and cell wall-related tolerance genes.
Sharma et al. (2020)	Citronellol	Purchased directly from Sigma-Aldrich	*C. albicans*	Inhibitory effect on the secretion of extracellular phospholipases and proteinases and biofilm formation.
Xu et al. (2019)	Coumarin	Purchased directly from Sangon Biotech Co., Ltd.	*C. albicans*	Inhibited fungal adhesion and biofilm formation; additionally, destroyed preformed biofilm.
Behbehani et al. (2019)	Epigallocatechin gallate	Purchased directly from Sigma-Aldrich	Various *Candida* species	The minimum biofilm inhibitory concentration (MBIC) range of this compound was lower than fluconazole and ketoconazole.
Ning et al. (2015)	Epigallocatechin gallate	Purchased directly from Sigma-Aldrich	*C. parapsilosis*, *C. krusei*, *C. tropicalis*, *C. kefyr*, *C. glabrata*, and *C. albicans*	A synergism effect was reported between this compound and fluconazole, miconazole, and AMB against the biofilm community of various *Candida* species.
Evensen and Braun (2009)	Epigallocatechin gallate	NR	*C. albicans*	Reduced 75% of viable cells during biofilm development.
Chauhan et al. (2011)	Ethyl alcohol	NR	*C. albicans*	Inhibited biofilm development and germ tube formation.
Mishra et al. (2021b)	Eucalyptol	Purchased directly from Sigma-Aldrich	*C. albicans* and *C. glabrata*	Eucalyptol/β-cyclodextrin inclusion complex to gellan/polyvinyl alcohol nanofibers suppressed 70% biofilm of fungi.
Gupta et al. (2021b)	Eucalyptol	Purchased directly from Sigma-Aldrich	*C. albicans* and *C. glabrata*	Showed antibiofilm activity against mature biofilm.
Müller-Sepúlveda et al. (2020)	Eucalyptol	*Lavandula dentata* L EO	*C. albicans*	Suppressed adhesion, morphogenesis, biofilm formation, altered microarchitecture, and reduced the viability of the established biofilm.
Manoharan et al. (2017b)	Fenchone	Purchased directly from Sigma-Aldrich	*C. albicans*	Decreased biofilm formation.
Manoharan et al. (2017b)	Fenchyl alcohol	Purchased directly from Sigma-Aldrich	*C. albicans*	Reduced biofilm formation and hyphal formation.
Alves-Silva et al. (2016)	Geranyl acetate	*Daucus carota* subsp*. carota*	*C. albicans*	Decreased biofilm biomass and cell viability.
Thakre et al. (2018)	Limonene	Purchased directly from Sigma-Aldrich	*C. albicans*	This compound was more effective against adhesion followed by the development and maturation of biofilm; additionally, it showed synergy with fluconazole against biofilm growth.
Raut et al. (2013)	Linalool	Purchased directly from Sigma-Aldrich	*C. albicans*	Suppressed yeast-to-hypha dimorphism and biofilm formation.
Kim et al. (2020)	Linoleic acid	Purchased directly from Sigma-Aldrich	Fluconazole-resistant *C. albicans* (*S. aureus*)	Inhibited biofilm formation, hyphal growth, and cell aggregation by *C. albicans*. Additionally, linoleic acid suppressed mixed *S. aureus* and *C. albicans* biofilms.
Barad et al. (2017)	linoleic acid	Purchased directly from Sigma-Aldrich	*C. albicans*	The Zinc oxide NPs coated with Chitosan-linoleic acid inhibited *C. albicans* biofilm formation even better than fluconazole.
Suchodolski et al. (2021)	Menthol	Purchased directly from Sigma-Aldrich	*C. krusei*, *C. albicans*, *C. glabrata*, and *C. parapsilosis*	Imidazolium ionic liquids based on (-)-menthol inhibited biofilm formation.
Saharkhiz et al. (2012)	Menthol	*Mentha piperita* L EO	*C. dubliniensis* and *C. albicans*	Inhibited biofilm development.
Raut et al. (2013)	Nerol	Purchased directly from Sigma-Aldrich	*C. albicans*	Inhibited biofilm formation.
Li et al. (2017)	Osthole (a natural coumarin)	Purchased directly from national Institutes for Food and Drug Control, Beijing, China	Fluconazole-resistant *C. albicans*	The results indicated synergism of osthole and fluconazole.
Ferreira et al. (2021)	P-coumaric acid	Purchased directly from Sigma-Aldrich	*C. albicans*, *C. glabrata* and *C. krusei*	p-coumaric acid-loaded liquid crystalline systems exhibited higher elimination of established biofilms than AMB and fluconazole.
Liu et al. (2021)	Phloretin	Purchased directly from Aladdin	*C. albicans*	Suppressed biofilm formation and the yeast-to-hypha transition.
Priya et al. (2021a)	Piperine	Purchased directly from HiMedia	*C. albicans*	Treatment of *Candida* biofilm by thymol and piperine leads to the synergistic effect; additionally, it reduces *Candida* attachment and hyphal extension.
Thakre et al. (2021)	Piperine	Purchased directly from Sigma-Aldrich	Fluconazole-resistant *C. albicans*	Indicated good synergistic activity with fluconazole against the biofilm community.
Priya and Pandian (2020)	Piperine	Purchased directly from HiMedia	*C. albicans*	Suppressed biofilm and hyphal morphogenesis.
Shahzad et al. (2014)	Pyrogallol	NR	*C. albicans*	Indicated antibiofilm activity.
Chevalier et al. (2012)	Saponin	*Solidago virgaurea*	*C. albicans*	Yeast-to-hypha transition phase, biofilm formation, and established biofilms were strongly suppressed.
Sadowska et al. (2014)	Saponin	*Medicago sativa* and *Saponaria officinalis*	*C. albicans*	This compound inhibited hyphal growth, yeast attachment, germ tube formation, and biofilm formation.
Yang et al. (2018)	Saponin	Rhizomes of *Dioscorea panthaica* Prain et Burk	*C. albicans*	Inhibited biofilm formation, adhesion, yeast-to-hyphal transition phase, and phospholipase production. Additionally, this compound led to the production of endogenous ROS, consequently disrupting the cell membrane in planktonic cells.
Coleman et al. (2010)	Saponin	From various natural products	*C. albicans*	Disrupted hyphae and biofilm formation.
Lemos et al. (2020)	Scopoletin (a natural coumarin)	*Mitracarpus frigidus*	MDR *C. tropicalis*	Suppressed formation of elongated fungal forms, the growth rate of established biofilms, and biofilms formation on the surface of coverslips.
Morey et al. (2016)	Tannins	*Stryphnodendron adstringens*	*C. tropicalis*	Decreased biofilm biomass.
Manoharan et al. (2017a)	α-longipinene	NR	*C. albicans*	Inhibited biofilm formation and showed synergistic effect with linalool.
Zuzarte et al. (2021)	α-pinene and β–pinene	*Bupleurum* subsp. *paniculatum* (Brot.) H. Wolff EO	*C. albicans* (*Cryptococcus neoformans* and other dermatophytes)	Inhibited germ tube formation and eliminated mature biofilm.
Rivas Da Silva et al. (2012)	α-pinene and β–pinene	Purchased directly from Sigma-Aldrich	*C. albicans* (various bacterial strains)	These compounds were highly toxic to *C. albicans* and prevented biofilm formation by this fungus*.*
Ramage et al. (2012)	α-terpineol	Purchased directly from Sigma-Aldrich	*C. albicans*	Showed rapid antibiofilm activity.
Alves-Silva et al. (219)	β–pinene	*Santolina impressa*	*C. albicans* (*C. neoformans*, *Epidermophyton floccosum* and *Trichophytum rubrum*)	Inhibited germ tube formation and eliminated mature biofilm.

EO, essential oil; NPs, nanoparticles; ROS, reactive oxygen species; NR, not reported; AMB, amphotericin B; MDR, multidrug-resistant.

## Conclusion

This article discussed various natural compounds with the potential for inhibiting and degrading *Candida* biofilm. These compounds have shown negative effects on different cellular mechanisms of *Candida* and could boost the efficacy of antifungals against biofilm communities. Thus, natural compounds should be considered for the treatment of *Candida* biofilm-associated infections, especially for established oropharyngeal candidiasis, since local treatment is easier in these infections. Disadvantages such as low oral bioavailability, water insolubility, and rapid metabolism and degradation limit the clinical application of many natural compounds. The use of various drug delivery platforms could be useful in such cases and should be evaluated in future studies. Most studies have evaluated the antibiofilm efficacy of natural compounds only in *C. albicans*, and that also *in vitro*. As such, future studies should evaluate the antibiofilm activity of natural compounds against other clinical important clinically important species such as *Candida auris*. Furthermore, before major clinical usage of natural compounds, more animal studies and clinical trials are required to confirm *in vitro* findings.

## Abbreviations

EOs, essential oils; ALS, agglutinin‐like sequence; PEG, polyethylene glycol; NPs, nanoparticles; GC-GC/MS, gas chromatography-mass spectrometry; MICs, minimum inhibitory concentrations; PLA, poly (lactic acid); AMB, amphotericin B; PMMA, polymerized Polymethyl Methacrylate; TTO, tea tree oil; LCS, liquid crystalline system; GA, electrospun gellan; PVA, polyvinyl alcohol; CM-ML, cinnamaldehyde in a multilamellar liposome; PLGA, poly (DL-lactide-co-glycolide).
